# Construction of a ubiquitination-related gene signature to predict prognosis and treatment response in lung adenocarcinoma

**DOI:** 10.12669/pjms.42.3.13747

**Published:** 2026-03

**Authors:** Xue Li, Jianian Lai, Meifang Guo, Zhen Lian

**Affiliations:** 1 Xue Li Department of Radiation Oncology, Tianjin Medical University Cancer Institute and Hospital, National Clinical Research Center for Cancer, Key Laboratory of Cancer Prevention and Therapy, Tianjin’s Clinical Research Center for Cancer, Tianjin 300060, China; 2 Jianian Lai Department of Radiation Oncology, Tianjin Medical University Cancer Institute and Hospital, National Clinical Research Center for Cancer, Key Laboratory of Cancer Prevention and Therapy, Tianjin’s Clinical Research Center for Cancer, Tianjin 300060, China; 3 Meifang Guo Department of Ultrasonic Diagnosis, The Second People’s Hospital of Dongying, Dongying 257200, Shandong, China; 4 Zhen Lian Department of Emergency, Tianjin Medical University Cancer Institute and Hospital, National Clinical Research Center for Cancer, Key Laboratory of Cancer Prevention and Therapy, Tianjin’s Clinical Research Center for Cancer, Tianjin 300060, China

**Keywords:** Cancer immunity, Lung adenocarcinoma, Prognosis, Ubiquitination

## Abstract

**Background & Objective:**

The prognosis of lung adenocarcinoma (LUAD) remains poor, primarily due to treatment resistance and a lack of effective biomarkers. This research investigated the potential of ubiquitination-related genes (UbRG) as prognostic indicators for LUAD.

**Methodology:**

This study was conducted at Tianjin Medical University Cancer Institute and Hospital from May 2025 to October 2025. Leveraging data from TCGA, we initially employed WGCNA to identify key modules of UbRG associated with LUAD. Then, a multigene prognostic signature was developed. To investigate the mechanisms, GSEA and CIBERSORT were used to detect activated signaling pathways and immune landscape, respectively. Finally, drug sensitivity analysis was performed.

**Results:**

Our analysis identified nine gene pairs that constitute the UbRG prognostic signature, with individuals in the high-risk cohort generally experiencing worse outcomes. Pathway enrichment analyses revealed immune response pathways in the low-risk cohort and the cell cycle and DNA replication pathways in the high-risk cohort. CIBERSORT revealed distinct immune cell distributions, with more CD4^+^ T cells and DCs in the low-risk cohort. Drug sensitivity analysis suggest that the low-risk group may exhibit a greater sensitivity to both chemotherapy and targeted treatments.

**Conclusion:**

This study presents a novel UbRG signature for LUAD prognosis, emphasizing the role of ubiquitination in the immune response. These findings may guide future therapeutic strategies in LUAD.

## INTRODUCTION

Lung cancer remains the leading cause of cancer-related fatalities worldwide. Over 85% of lung cancer cases are classified as non-small cell lung cancer (NSCLC), with lung adenocarcinoma (LUAD) being the most prevalent subtype.^1^ Treatment advances have revolutionized the paradigms of therapy for LUAD patients.^2^,^3^ However, most patients do not benefit from these therapeutic approaches or obtain long-lasting benefits^4^,^5^ and the underlying mechanisms are still unclear.

Ubiquitination, a well-established form of protein modification, is a key player in critical biological functions, such as protein degradation, cell signal transduction, DNA damage repair, the transport and endocytosis of membrane proteins, and immune responses.^6^,^7^ A growing body of evidence indicates that ubiquitination has a role in tumor progression and therapeutic resistance.^8^ Numerous studies have explored the potential of the ubiquitylation system as a therapeutic target and biomarker in cancer.^9^,^10^ Nevertheless, its function in LUAD is still not well understood.

**Supplementary Fig.1:**
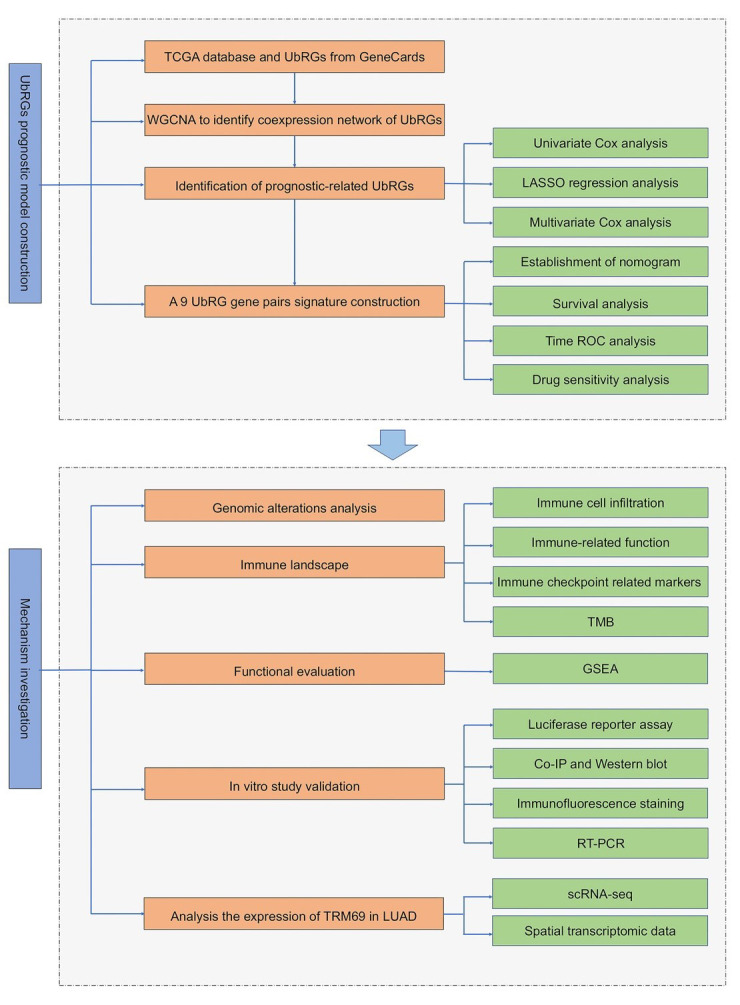
Flow chart showing the overall design of this study.

To clarify the function of the ubiquitination system in LUAD, we first screened for ubiquitination-related genes (UbRG) associated with the outcomes of LUAD patients. These UbRG were subsequently used to establish a risk score signature, and a nomogram was further developed by combining clinicopathological parameters and risk scores. To explore the potential underlying mechanisms, we investigated the activated signaling pathways and immune landscape in different risk groups. The analysis procedures are shown in Supplementary Fig.1.

## METHODOLOGY

This study was conducted at Tianjin Medical University Cancer Institute and Hospital from May 2025 to October 2025. The Cancer Genome Atlas (TCGA) database (TCGA-LUAD, 2025.05.01) was used to gather gene expression data (FPKM), somatic mutation data and clinical data for LUAD tumor samples. Additionally, 1393 ubiquitination-related genes were extracted from the GeneCards database.

### Ethics approval and consent to participate:

Not applicable.

### WGCNA:

To identify hub gene modules that are strongly linked to ubiquitination in patients with LUAD, the “WGCNA” package was used. First, network modules and coexpressed gene modules were generated via topological overlap measurement (TOM) and the dynamic hybrid cutting method, respectively. The modules were subsequently merged on the basis of gene similarity. Finally, we assessed the module significance (MS) and gene significance (GS) to determine the relationships between genes and modules. Ultimately, modules that were strongly related to survival were screened for additional studies.

### Development and validation of the UbRG Signatures:

Initially, we systematically paired genes within the hub module, establishing ubiquitination-related gene pairs (UbRGP). Next, we evaluated the relative expression levels of each gene pair to assign a scoring system. In cases where the level of the first gene was higher than that of the second within the same sample, the UbRGP received a score of 1; otherwise, they received a score of 0. Using Cox regression, we pinpointed the UbRGP with significant survival predictive power. To refine our model, we applied LASSO analysis, deriving a risk score signature on the basis of the weighted sum of each gene’s expression multiplied by its respective coefficient. Finally, we stratified LUAD samples into low- and high-risk groups by dividing them according to the median risk score. With the R packages “survival” and “timeROC”, Kaplan-Meier curve and ROC analyses were performed to assess the reliability of this signature. The independence of this signature was subsequently assessed via Cox regression analysis, which utilized the “glmnet” and “survival” packages. A nomogram was created to assess the survival of LUAD patients, employing the R packages “regplot”, “survival”, and “rms”. The degree of agreement between the predicted and observed survival probabilities was quantified via the concordance index (C-index). A rating of one denotes perfect prediction.

### GSEA:

To identify key biological pathways and functional distinctions, we performed gene set enrichment analysis (GSEA) with the “enrichplot” and “clusterProfiler” packages.^11^ The gene set database used for enrichment was MSigDB. Statistical significance for filtering conditions was defined as *P* < 0.05.

### Immune profiles associated with the signature:

Initially, to evaluate the spatial arrangement of infiltrating immune cells, CIBERSORT was utilized.^12^ Tumor mutation burden (TMB) was quantified by dividing the aggregate number of genetic variants by the cumulative length of exons, a calculation automated via a Perl script.

### Drug sensitivity analysis:

Drug sensitivity analysis was performed using the R package “oncoPredict”. The IC50 reference dataset used for prediction was derived from the Genomics of Drug Sensitivity in Cancer (GDSC) database. The IC50 scores between high- and low-risk groups were analyzed to predict the efficacy of chemotherapy drugs for LUAD patients.

### Statistical analysis:

Differential analysis and drug sensitivity analysis was conducted via a Wilcoxon log-rank test. Correlation analysis was carried out with Spearman’s test utilizing the R package “limma”. For survival analysis, we employed the Kaplan-Meier method with the “survminer” and “survival” packages and considered the results to be statistically significant when the log-rank test yielded *P* values less than 0.05. Cox regression was carried out via the “survival” package. Significance levels are denoted by “*”, “**”, and “***” for *P* values less than 0.05, 0.01, and 0.001, respectively. All data processing and statistical computations were carried out via R software, version 4.2.2.

## RESULTS

### Development of the UbRG prognostic signature:

**Fig.1:**
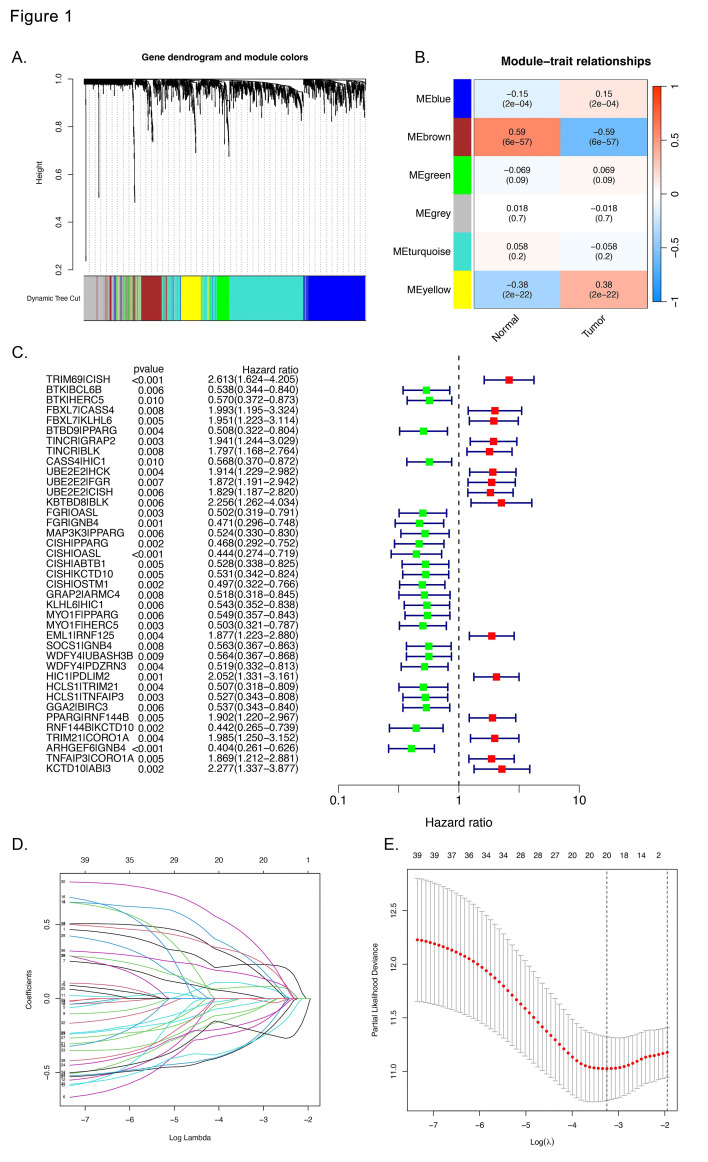
Development of the ubiquitination-related prognostic signature. **(A)** The gene dendrogram and module colors indicate that the UbRG are clustered into 6 categories. **(B)** Heatmap showing the relationships between merged coexpression modules and clinical features. **(C)** Univariate Cox regression analysis revealed 39 UbRGP with prognostic value. **(D, E)** LASSO Cox regression analysis was utilized to select genes appropriate for building the ideal model.

First, we employed WGCNA to identify the coexpression network of the UbRG in LUAD. The results revealed a clear division of the samples into six modules (Fig.1A-B). The brown module displayed a significant negative correlation with LUAD and was thus specifically examined for subsequent analyses. Then, we constructed UbRGP to eliminate the bias caused by varying measurements across platforms. From the 127 genes in the hub module, we identified 2043 valid UbRGP via an iterative process and a binary expression matrix. Univariate Cox regression analysis identified 39 prognostic UbRGP (Fig.1C). LASSO Cox regression was performed to remove overfitting genes. This process resulted in a final selection of 20 nonzero coefficients (Fig.1D-E). Finally, we used multivariate Cox regression analysis to identify nine gene pairs (TRIM69|CISH, FBXL7|CASS4, KBTBD8|BLK, GRAP2|ARMC4, MYO1F|PPARG, EML1|RNF125, HIC1|PDLIM2, HCLS1|TRIM21, and RNF144B|KCTD10) for the creation of the prognostic risk model.

### Construction and verification of a prognostic nomogram for LUAD:

**Fig.2:**
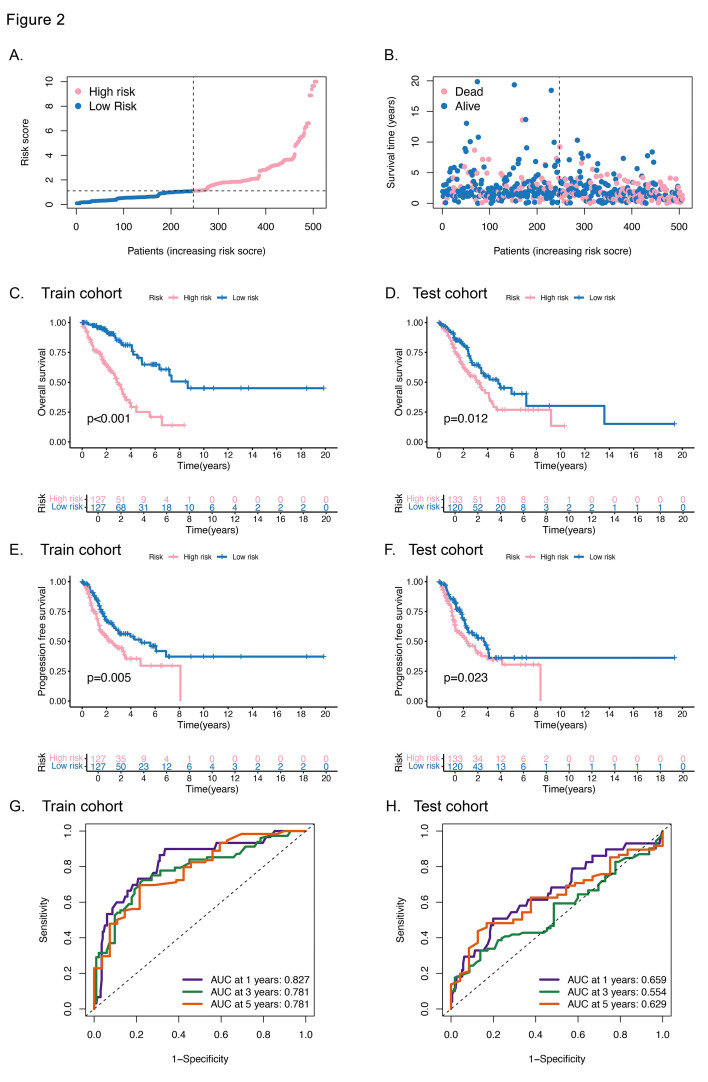
Efficacy validation of the ubiquitination-related prognostic signature. **(A)** Distribution of risk scores in the LUAD cohort. **(B)** Distribution of the corresponding risk score and survival status for each patient. **(C) and (D)** Overall survival of patients in the two risk groups in the training cohort and test cohort, respectively. **(E) and (F)** Progression-free survival of patients in the two-risk group in the training cohort and test cohort, respectively. **(G) and (H)** ROC curves illustrating the predictive efficacy of the signature for OS at 1, 3, and 5 years in different datasets.

Following the methodology described earlier, we employed the nine identified gene pairs to calculate risk scores. Our analysis revealed a clear trend: mortality rates increased steadily with increasing risk scores (Fig.2A-B). To evaluate the predictive power of the UbRG signature, we first partitioned the TCGA-LUAD dataset into training and testing subsets via the R package “caret”. The results of the Kaplan-Meier analysis demonstrated that individuals classified in the high-risk group, both in the training and testing subsets, presented a poorer prognosis than did those in the low-risk group (Fig.2C-F). Further validation through ROC analysis yielded AUC values of 0.827, 0.781, and 0.781 for 1-, 3-, and 5-year overall survival prediction, respectively, in the training set. The testing set produced slightly lower but still meaningful AUCs for the same time intervals (Fig.2G-H). These findings indicate that the UbRG signature serves as an effective model for prognostic prediction.

**Fig.3:**
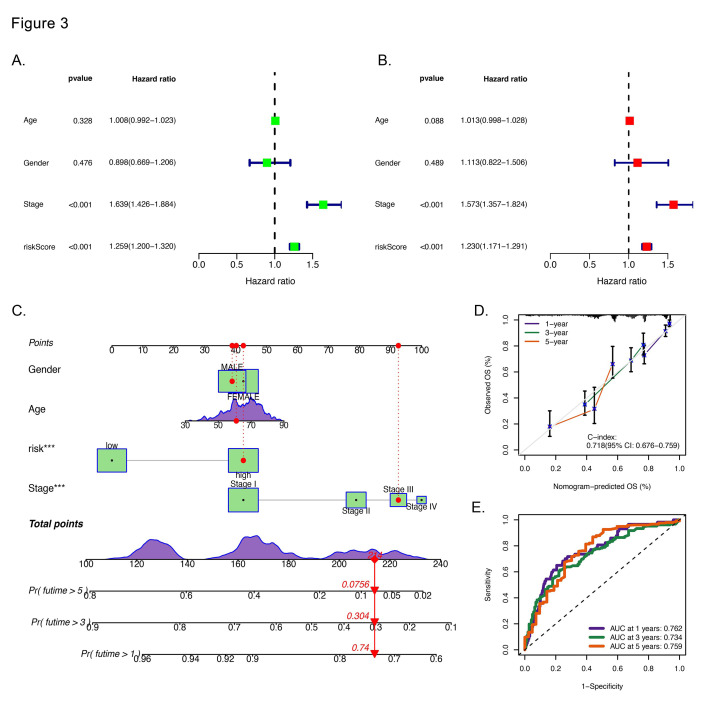
Construction and validation of a prognostic nomogram for LUAD. Univariate (A) and multivariate (B) Cox analyses demonstrated that the risk score is an independent prognostic factor. (C) The nomogram displays the survival predictive value of the risk score and clinical features. (D) The calibration curve illustrates the ideal predictive value of the nomogram. (E) ROC curves demonstrating the high predictive efficiency of the nomogram.

Additionally, we explored connections between the risk score and clinical characteristics. Univariate Cox regression yielded a significant hazard ratio of 1.259 (Fig.3A). In the multivariate analysis, the hazard ratio remained significant at 1.230 (Fig.3B). These results clearly suggest that this risk score is a robust standalone indicator for prognosis. Furthermore, we developed a nomogram to exploit the prognostic performance of the UbRG signature (Fig.3C). Calibration plot validation revealed the nomogram’s robust predictive accuracy (Fig.3D). Moreover, the AUC values for the one, three and five-years predictions were approximately 0.76, 0.73, and 0.76, respectively (Fig.3E). Taken together, these findings highlight the promise of this novel nomogram as an effective tool for predicting patient outcomes.

### Functional evaluation of the UbRG signature:

**Fig.4:**
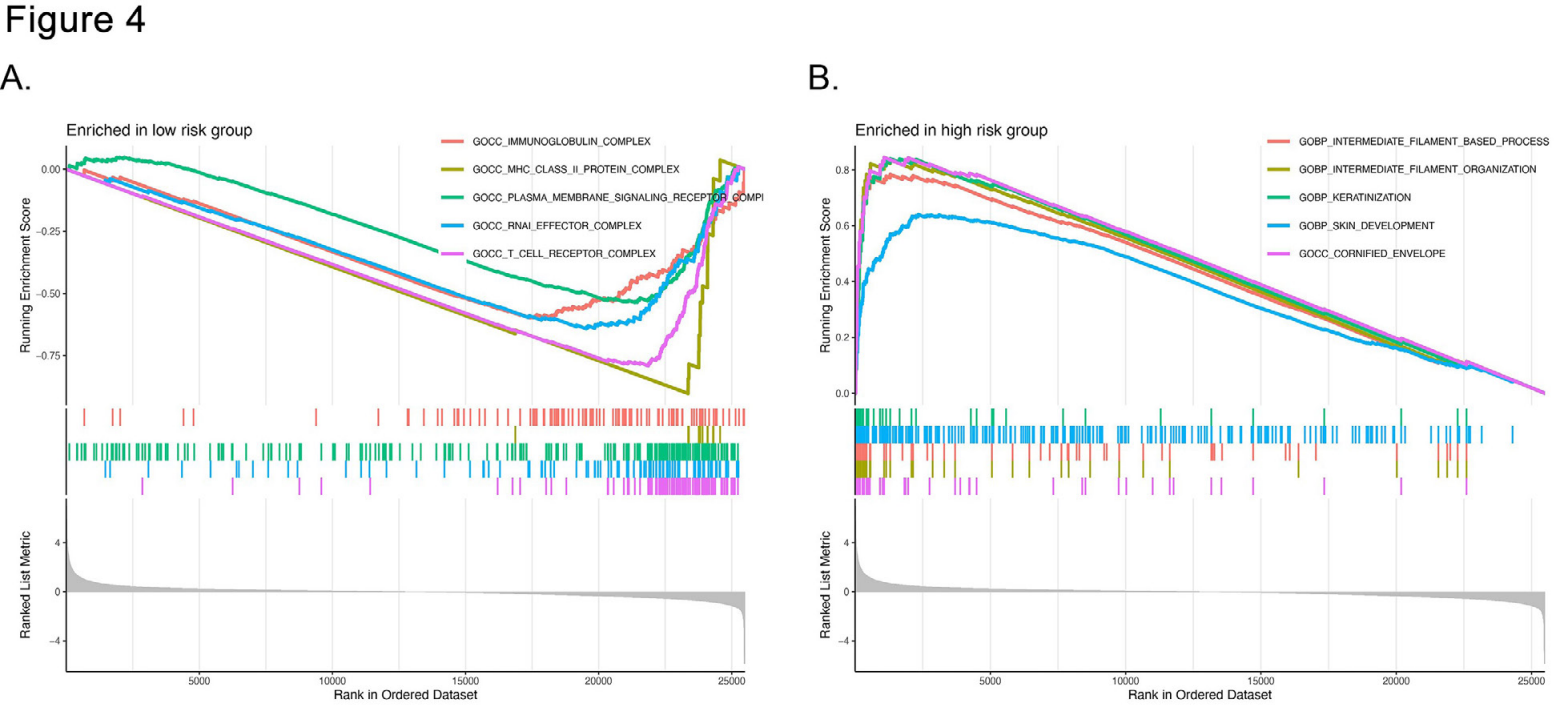
Gene set enrichment analysis of the UbRG signature in the high- and low-risk groups. (A) and (B) GO enrichment analyses of the genes in the low- and high-risk groups, respectively.

GSEA was conducted to investigate the underlying signaling mechanisms involved. Gene Ontology (GO) functional annotation revealed that biological processes in the low-risk group were markedly enriched for MHC class II protein complexes, immunoglobulin assemblies, plasma membrane receptor activity, RNA-mediated effector complexes, and T-cell receptor clusters (Fig.4A). In contrast, the high-risk group displayed pronounced enrichment in intermediate filament arrangement, keratinocyte differentiation, epidermal development, and cornified envelope assembly (Fig.4B).

### Immune landscape:

**Fig.5:**
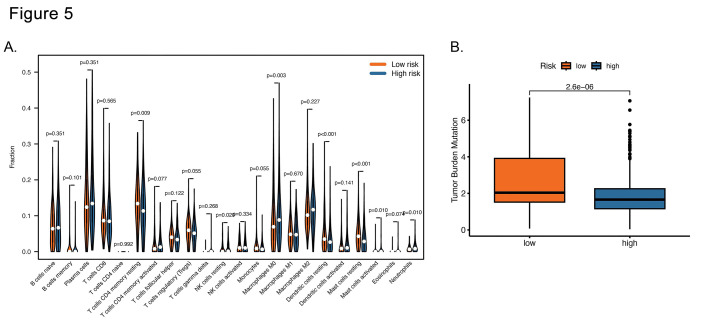
Immune landscape of the risk score model. (A) Analysis of variations in immune cell infiltration between the high-risk and low-risk groups with the CIBERSORT algorithm. (B) Comparison of TMB between the two groups.

Initially, we analyzed the distribution of immune cell infiltration in LUAD patients. As shown in Fig.5A, the low-risk set had notably higher counts of resting memory CD4 T cells, resting dendritic cells (DCs), and mast cells. In contrast, the high-risk group presented a greater presence of resting natural killer (NK) cells, M0 macrophages, and neutrophils. We also explored the correlation between TMB and the risk score and found that the low-risk group had a substantially greater TMB than their high-risk counterparts did (Fig.5B). Taken together, these observations imply that these UbRG may influence the prognosis of LUAD patients by modulating immune cell activity.

### Drug sensitivity analysis:

**Fig.6:**
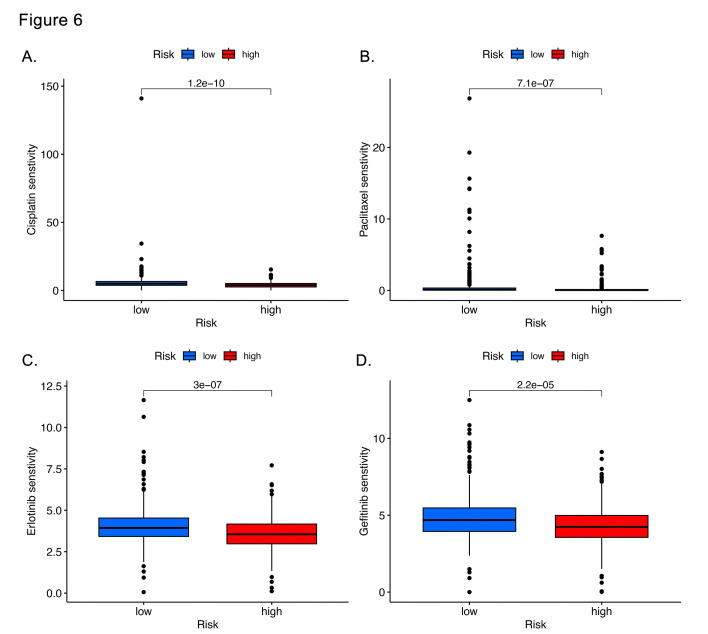
Drug sensitivity analysis. (A)Cisplatin. (B) Paclitaxel. (C) Erlotinib. (D) Gefitinib.

To investigate the predictive role of risk score in LUAD treatment, drug sensitivity analysis was performed. The results indicated that, the IC50 values were higher in the high-risk group compared to the low-risk group for most drugs, including chemotherapies like cisplatin and taxanes, as well as targeted therapies such as gefitinib and erlotinib (Fig.6A-E). These findings suggest that the low-risk group may exhibit a greater sensitivity to both chemotherapy and targeted treatments.

## DISCUSSION

In this study, we have developed a special 9-gene signature, which is related to ubiquitination and can predict the prognosis of LUAD patients. This signature performed well in both the training group and the verification group, and it was proved to be an independent predictor. In addition, we found that the high-risk and low-risk patient groups are obviously different in biological function, immune cell composition, and response to therapeutic drugs, which shows that ubiquitination has a complex impact on the development and therapeutic effect of LUAD.

Although significant progress has been made in the treatment of LUAD, such as surgery, chemotherapy, immunotherapy, and targeted drugs, patient outcomes remain poor.^13^ This is largely because tumors often develop resistance to treatment, and reliable biomarkers for early diagnosis and personalized therapy are still lacking.^14^-^16^ Given these challenges, uncovering the molecular drivers of LUAD and discovering new prognostic indicators are critical.^17^ Our research revealed the negative impact of UbRG on LUAD survival. Furthermore, a prognostic signature was developed using nine UbRGP. These results align with earlier studies. For instance, Zhang et al.^18^ developed a 7-UbRG signature for LUAD prognosis with an AUC of 0.73, which is slightly lower than our signature’s performance (AUC 0.827 in the training set). This discrepancy may be attributed to our use of gene pairs rather than individual genes, which reduces batch effect interference and improves cross-platform reproducibility. Clinically, the nomogram incorporating our UbRGP signature and clinicopathological parameters can be used to stratify LUAD patients into high- and low-risk groups, guiding treatment decisions. Furthermore, the identified UbRGP could serve as potential therapeutic targets.

Ubiquitination plays a pivotal role in regulating the immune response.^19^ Studies have shown that ubiquitination affects innate and adaptive immunity by regulating key proteins in different signaling pathways.^20^ In this study, the pathway enrichment analyses revealed distinct biological processes in different risk groups, with the low-risk group enriched in immune-related pathways and the high-risk group showing a propensity for the cell cycle and cancer-related pathways. Thus, we speculate that ubiquitination might regulate the prognosis of LUAD patients partially through antitumor immunity. In line with this, our findings revealed that the immune cell infiltration of the high-risk and low-risk groups differed significantly, characterized by a lower infiltration of Tregs and a higher concentration of memory resting CD4 T cells in the low-risk groups. These results indicate that the low-risk group had a more favorable immune microenvironment, which could correlate with improved patient survival and enhance the efficacy of immunotherapeutic strategies. Future studies should investigate the interplay between these UbRGP and various immune cell types, as well as their implications for immunotherapy.

### Limitations:

This study was conducted with data from the TCGA, and the results need to be confirmed with other datasets. Additionally, the datasets utilized may be subject to batch effects, which might affect the reproducibility of the identified prognostic markers. Furthermore, the relatively limited sample size restricts the ability to conduct comprehensive clinical validation.

## CONCLUSIONS

This study revealed a new ubiquitination-related gene signature that serves as a stable prognostic marker in LUAD. These findings may guide improving clinical risk stratification and personalized treatment decisions in LUAD. Furthermore, our research has identified connections between ubiquitination-related genes and LUAD immune pathways, potentially revealing new avenues for future lung cancer studies.

### Author’s contribution:

**XL:** Conceptualization, Funding acquisition, Investigation, Writing - review & editing, validation and responsible and accountable for the accuracy or integrity of the work.

**JL:** Data curation, Resources, Software, Supervision, Writing - original draft.

**MG:** Methodology, Project administration.

**ZL:** Formal analysis, Methodology, Visualization.

All authors have read and approved the final version of the manuscript. They are also accountable for the integrity of the study.
